# Supplementary figures and images for: Single cell heterogeneity in influenza A virus gene expression shapes the innate antiviral response to infection
Source: PLoS Pathog. 2020 Jul 2;16(7):e1008671. doi: 10.1371/journal.ppat.1008671 (PMC7363107; doi:10.1371/journal.ppat.1008671)

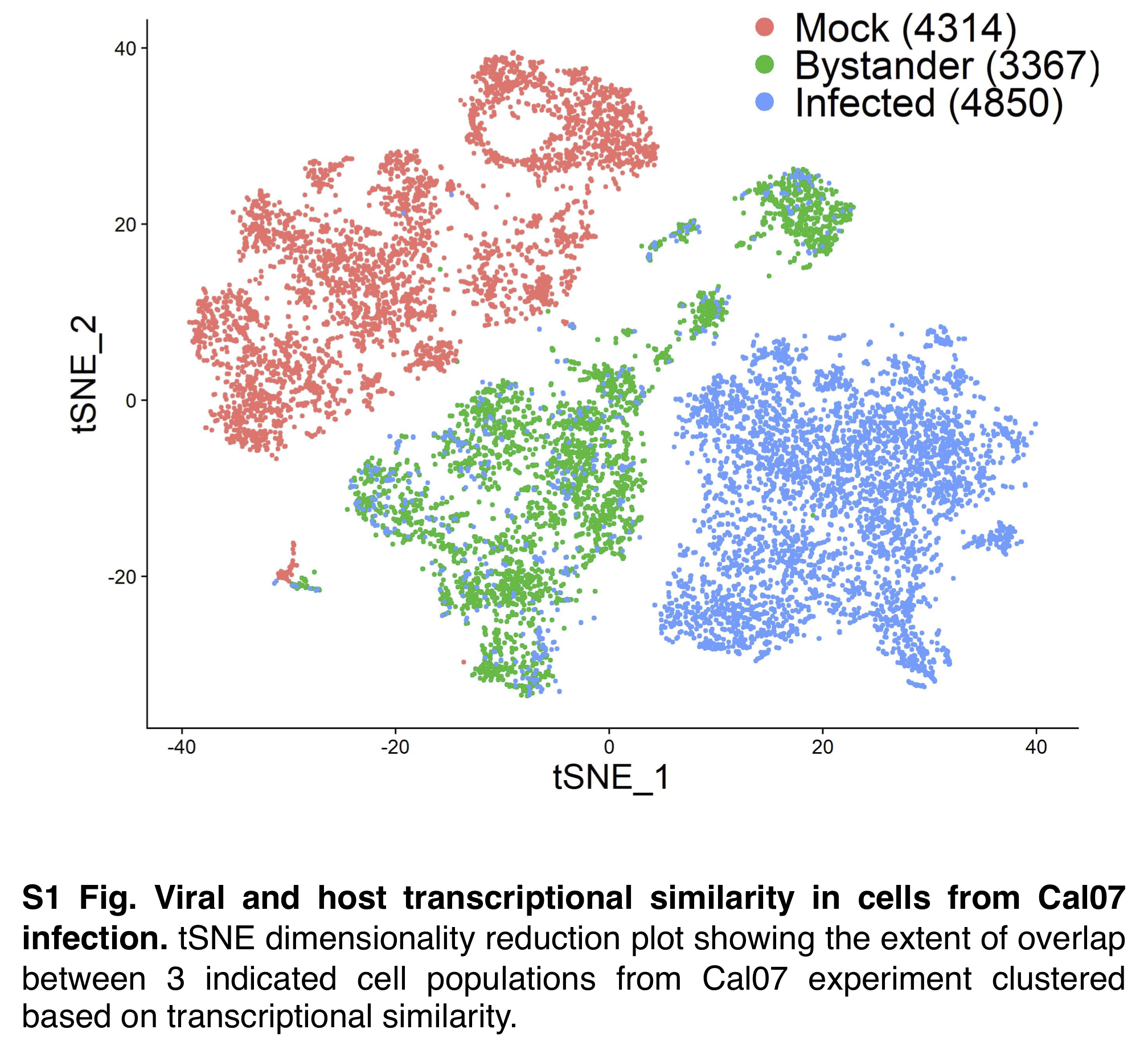

Supplement: S1 Fig — tSNE dimensionality reduction plot showing the extent of overlap between 3 indicated cell populations from Cal07 experiment clustered based on transcriptional similarity. (TIFF) [file ppat.1008671.s001.tiff]

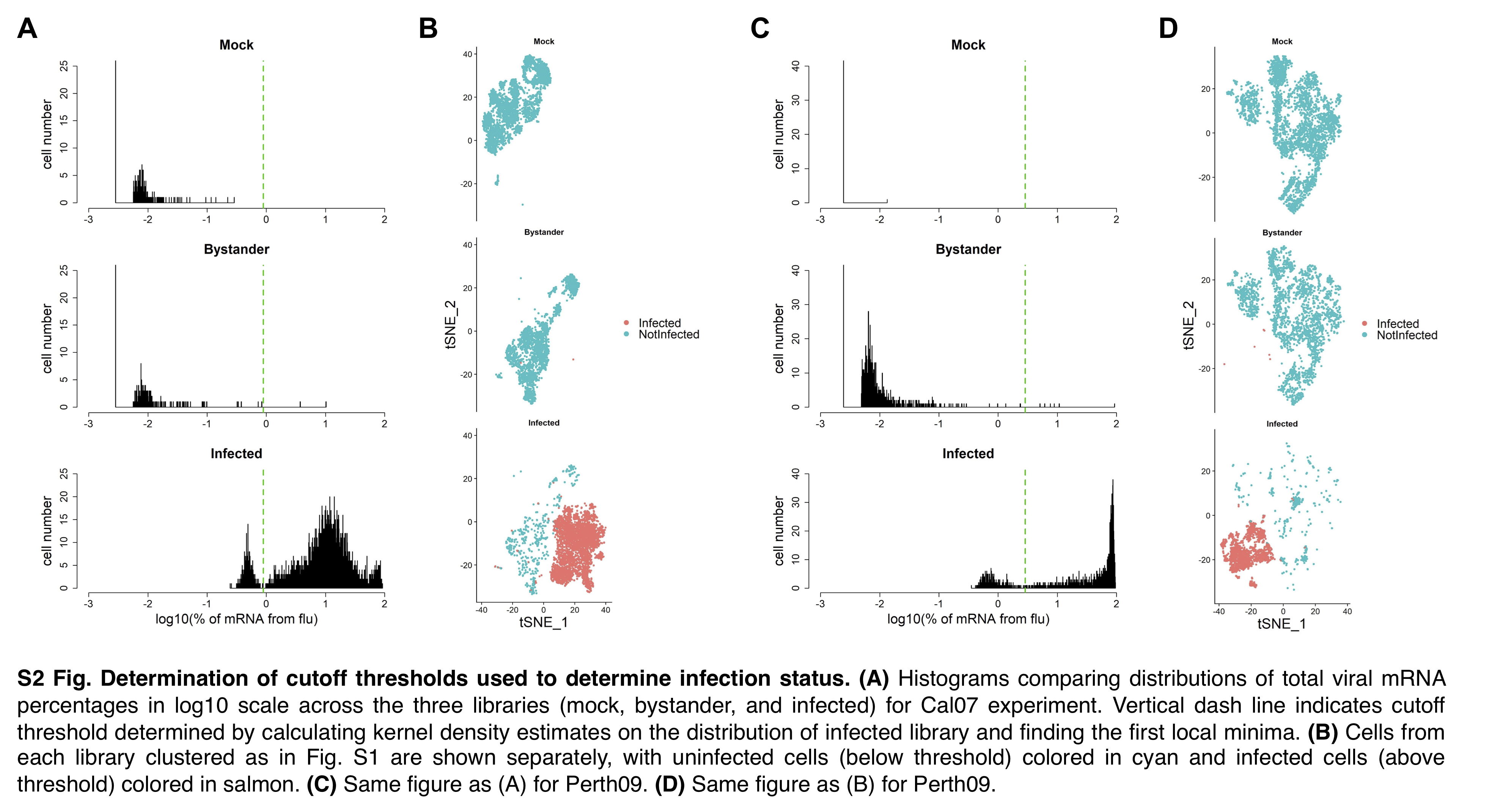

Supplement: S2 Fig — (A) Histograms comparing distributions of total viral mRNA percentages in log10 scale across the three libraries (mock, bystander, and infected) for Cal07 experiment. Vertical dash line indicates cutoff threshold determined by calculating kernel density estimates on the distribution of infected library and finding the first local minima. (B) Cells from each library clustered as in S1 Fig are shown separately, with uninfected cells (below threshold) colored in cyan and infected cells (above threshold) colored in salmon. (C) Same figure as (A) for Perth09. (D) Same figure as (B) for Perth09. (TIFF) [file ppat.1008671.s002.tiff]

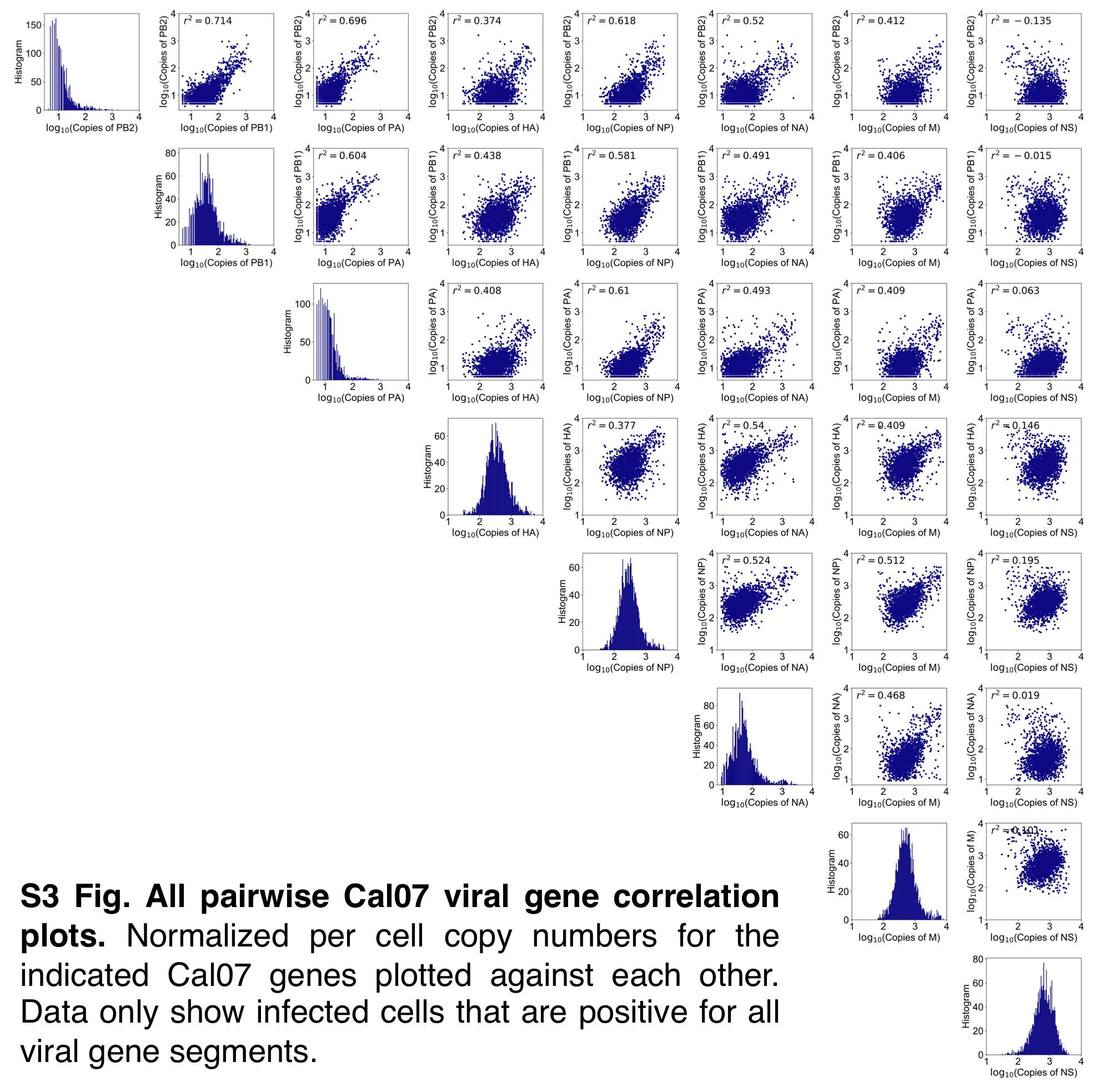

Supplement: S3 Fig — Normalized per cell copy numbers for the indicated Cal07 genes plotted against each other. Data only show infected cells that are positive for all viral gene segments. (TIFF) [file ppat.1008671.s003.tiff]

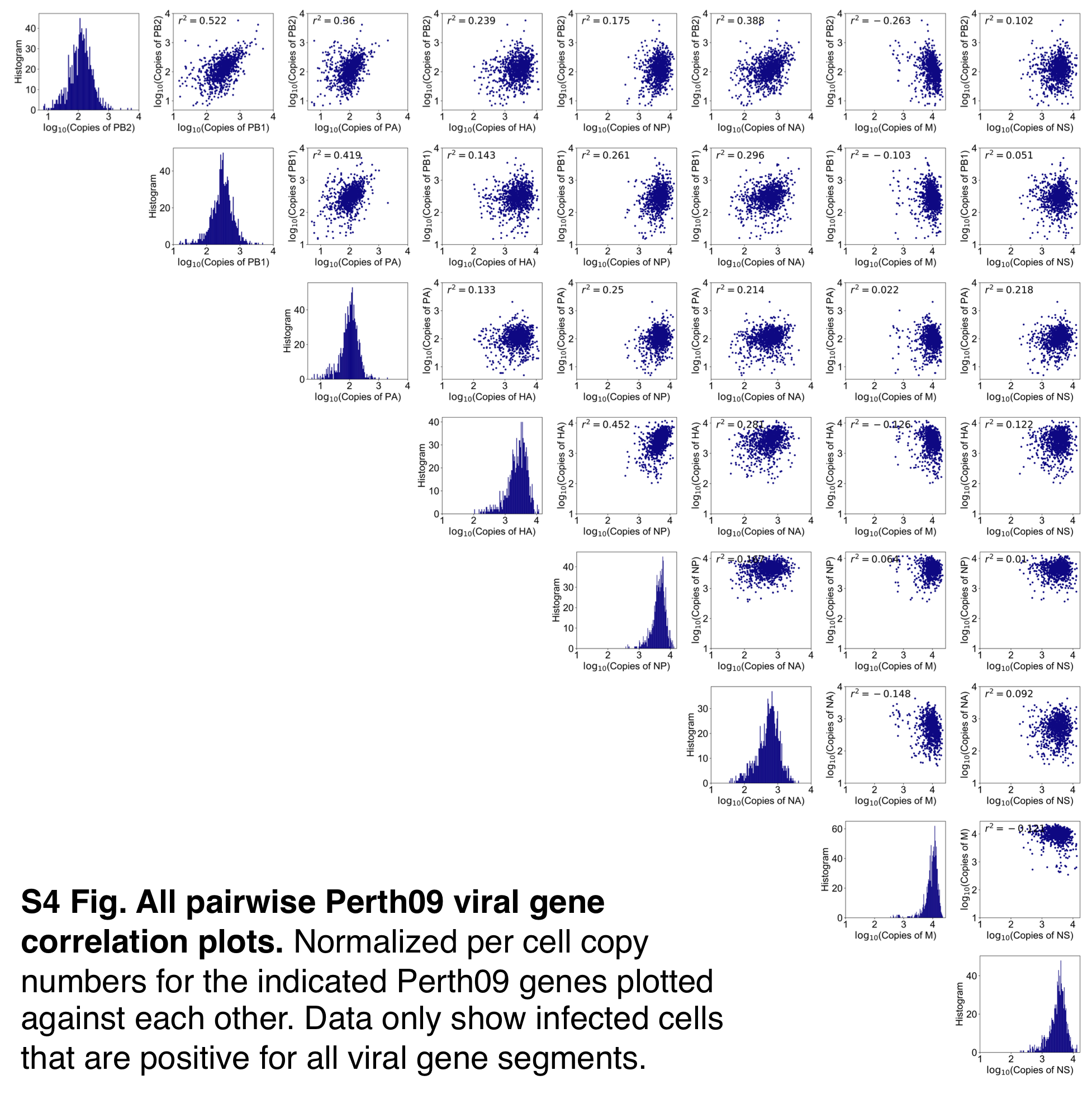

Supplement: S4 Fig — Normalized per cell copy numbers for the indicated Perth09 genes plotted against each other. Data only show infected cells that are positive for all viral gene segments. (TIFF) [file ppat.1008671.s004.tiff]

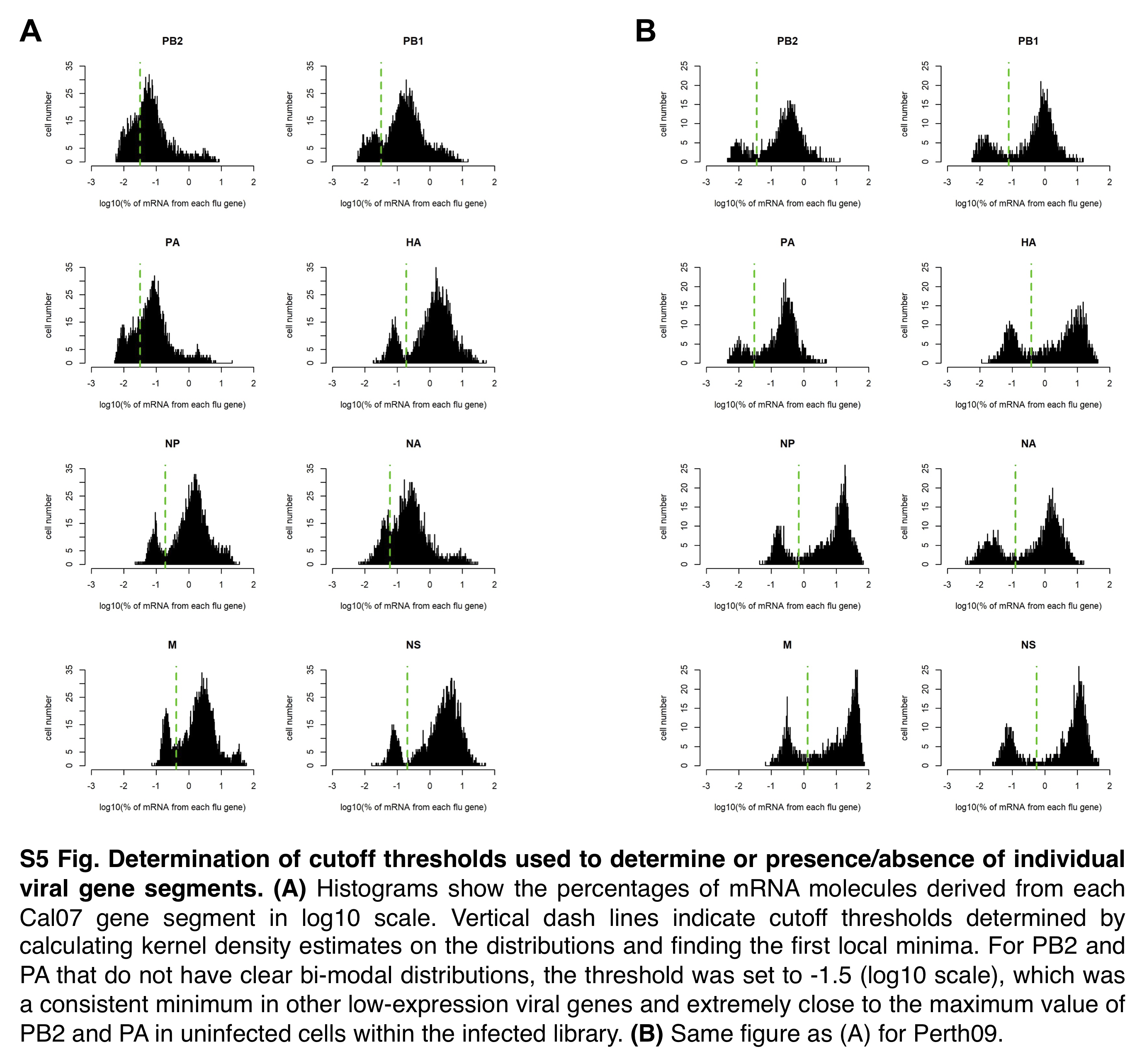

Supplement: S5 Fig — (A) Histograms show the percentages of mRNA molecules derived from each Cal07 gene segment in log10 scale. Vertical dash lines indicate cutoff thresholds determined by calculating kernel density estimates on the distributions and finding the first local minima. For PB2 and PA that do not have clear bi-modal distributions, the threshold was set to -1.5 (log10 scale), which was a consistent minimum in other low-expression viral genes and extremely close to the maximum value of PB2 and PA in uninfected cells within the infected library. (B) Same figure as (A) for Perth09. (TIFF) [file ppat.1008671.s005.tiff]

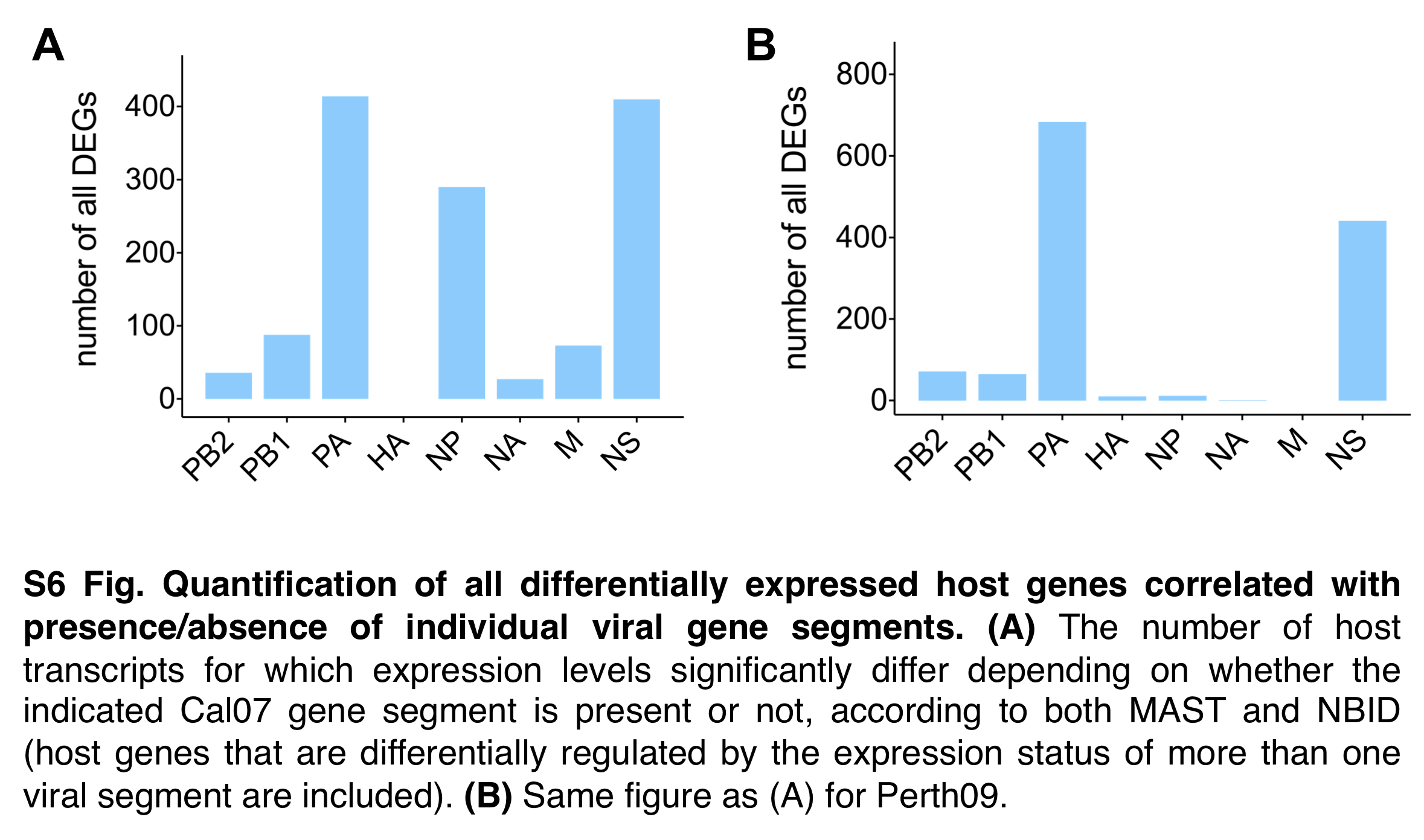

Supplement: S6 Fig — (A) The number of host transcripts for which expression levels significantly differ depending on whether the indicated Cal07 gene segment is present or not, according to both MAST and NBID (host genes that are differentially regulated by the expression status of more than one viral segment are included). (B) Same figure as (A) for Perth09. (TIFF) [file ppat.1008671.s006.tiff]

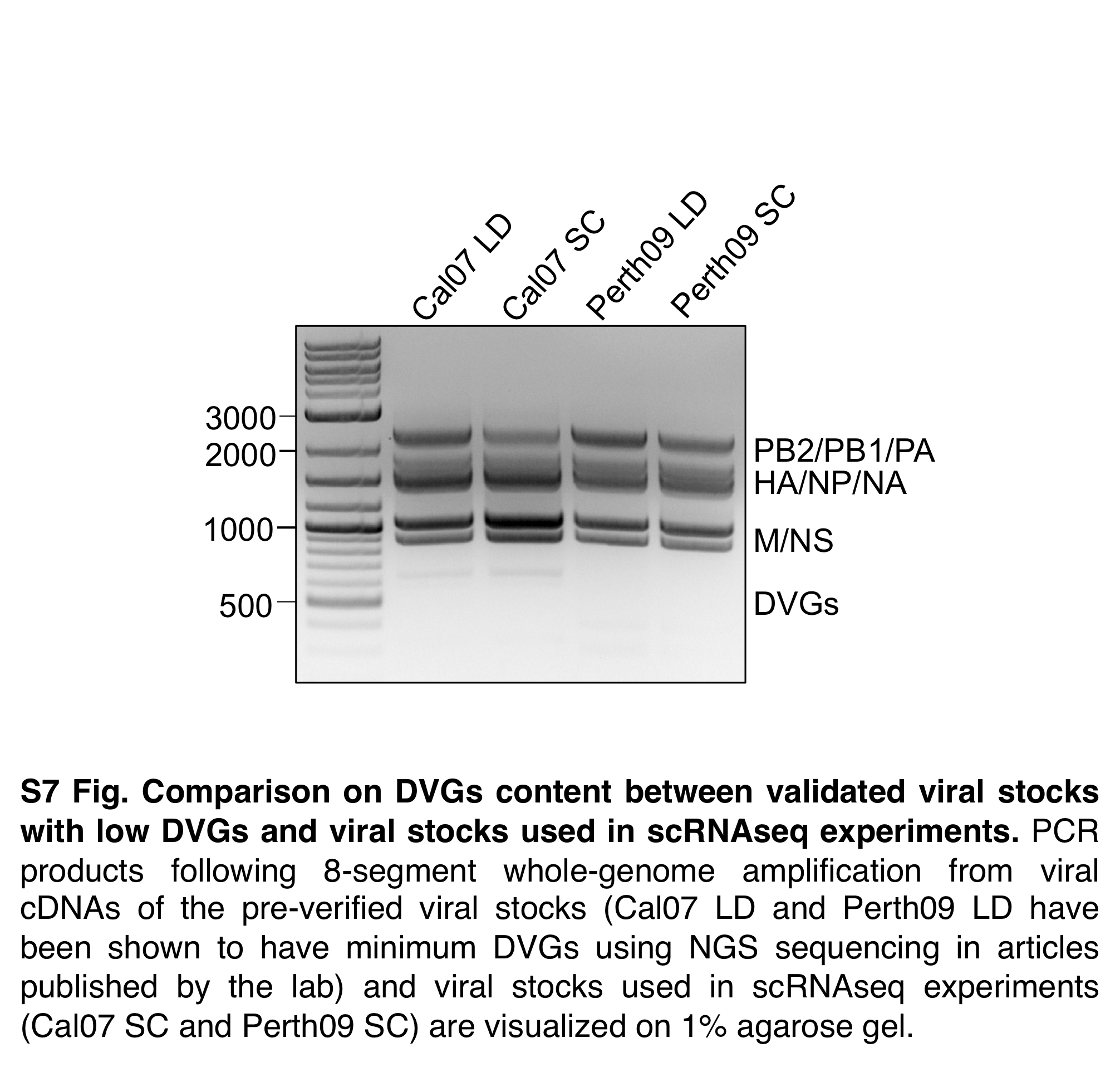

Supplement: S7 Fig — PCR products following 8-segment whole-genome amplification from viral cDNAs of the pre-verified viral stocks (Cal07 LD and Perth09 LD have been shown to have minimum DVGs using NGS sequencing in articles published by the lab) and viral stocks used in scRNAseq experiments (Cal07 SC and Perth09 SC) are visualized on 1% agarose gel. (TIFF) [file ppat.1008671.s007.tiff]
